# Characterizing the Diffusion and Rheological Properties of Aged Asphalt Binder Rejuvenated with Bio-Oil Based on Molecular Dynamic Simulations and Laboratory Experimentations

**DOI:** 10.3390/molecules26237080

**Published:** 2021-11-23

**Authors:** Xiaorui Zhang, Chao Han, Xinxing Zhou, Frédéric Otto, Fan Zhang

**Affiliations:** 1 School of Transportation, Southeast University, Nanjing 211189, China; 101004978@seu.edu.cn; 2 Fangshan R&D Base, Jsti Group, Nanjing 210017, China; hc1527@jsti.com; 3 Key Laboratory of Highway Construction and Maintenance Technology in Loess Region, Shanxi Transportation Technology Research & Development Co., Ltd., Taiyuan 030032, China; zxx09432338@whut.edu.cn; 4 State Key Laboratory of Silicate Materials for Architectures, Wuhan University of Technology, Wuhan 430070, China; 5 Chair and Institute of Highway Engineering, Mies-van-der-Rohe-Straße 1, 52074 Aachen, Germany; otto@isac.rwth-aachen.de

**Keywords:** aged asphalt binder, bio-oil, soybean, diffusion, rheological properties

## Abstract

Soybean-derived bio-oil is one of the vegetable-based oils that is gaining the most interest for potential use in the rejuvenation of aged asphalt binders. This laboratory study was conducted to characterize and quantify the diffusion and rheological properties of bio-oil-rejuvenated aged asphalt binder (BRAA) using soybean oil. In the study, the chemical structure of the soybean oil was comparatively characterized using an element analyzer (EA), gel permeation chromatography (GPC), and a Fourier infrared (FTIR) spectrometer, respectively. Based on the chemical structure of the bio-oil, BRAA molecular models were built for computing the diffusion parameters using molecular dynamic simulations. Likewise, a dynamic shear rheometer (DSR) test device was used for measuring and quantifying the rheological properties of the aged asphalt binder rejuvenated with 0%, 1%, 2%, 3%, 4%, and 5% soybean oil, respectively. The laboratory test results indicate that bio-oil could potentially improve the diffusion coefficients and phase angle of the aged asphalt binder. Similarly, the corresponding decrease in the complex shear modulus has a positive effect on the low-temperature properties of BRAA. For a bio-oil dosage 4.0%, the diffusion coefficients of the BRAA components are 1.52 × 10^−8^, 1.33 × 10^−8^, 3.47 × 10^−8^, 4.82 × 10^−8^ and 3.92 × 10^−8^, respectively. Similarly, the corresponding reduction in the complex shear modulus from 1.27 × 10^7^ Pa to 4.0 × 10^5^ Pa suggests an improvement in the low-temperature properties of BRAA. Overall, the study contributes to the literature on the potential use of soybean-derived bio-oil as a rejuvenator of aged asphalt binders.

## 1. Introduction

More than 80% of highways around the world are composed of asphalt pavements. Some of the characteristic properties and advantages contributing to the wide usage of asphalt pavements include good skidding and wearing resistance, driving comfortability, low noise, easy maintainability, and the deformation adaptability of the subgrade [1,2,3]. When in service, however, the asphalt binder within asphalt pavements ages as a function of time due to exposure to varying traffic loads and fluctuating environmental conditions, which is an undesired phenomenon [4,5].

A lot of research has been carried out on the physical and rheological characteristics of aged asphalt binders. It is believed that different asphalt binders have different anti-aging properties; however, the aging mechanism is essentially the same. For example, as the aging time of the asphalt binder increases, the penetration decreases, the penetration index increases, the softening point increases, the ductility decreases, etc. [6]. Likewise, the viscosity, complex shear modulus (G*) and creep stiffness (*S*) of the asphalt binder will typically increase as a function of aging time, with a decreasing trend in the phase angle (δ) and unrecoverable creep compliance (*J*_nr_) properties [7,8,9,10]. Ultimately, this shows how aging leads to an undesirable increase in the stiffness and embrittlement of the asphalt binder, which inevitably worsens the asphalt pavement’s performance, with manifestations of distresses such cracking, moisture damage, aggregate raveling, etc. [11,12]. Therefore, asphalt pavements would typically require re-surfacing, overlaying, maintenance, and/or rehabilitation during their service lives to restore their properties and performance characteristics [13]. In the case of aged asphalt binders and as part of the pavement maintenance/rehabilitation scheme, rejuvenators are often used for rejuvenating, restoring, and recycling the aged asphalt binder, including reclaimed asphalt pavement (RAP) and sealing materials [14,15].

Rejuvenated asphalt binder using residual soybean oil can be as good as, or even better than, the traditional mineral oil rejuvenators [16]. Data from relevant research institutions show that 6030.8 million tons of soybeans were produced in 2020 around the world. The growth data indicate the wide sources of soybean oil. As a result, if soybean oil waste can be promoted for use in recycling applications, it can be a viable alternative, and utilized to effectively reduce the cost of asphalt pavement regeneration technologies. The rejuvenators used as additives to rejuvenate oxidized asphalt binders are produced from aromatic hydrocarbon oils, which are mostly petroleum products. However, this type of oil contains a high proportion of carcinogenic polycyclic aromatic hydrocarbons (PAHs), and it easily volatilizes during the mixing and construction processes [17]. With the increasing awareness of environmental and public health concerns, it is possible that the use of base oils as a rejuvenator of traditional PAHs will be restricted or even eliminated. Environmentally friendly renewable materials may become a substitute for traditional petrochemical products.

The use of bio-oil as a bio-binder has the advantages of low volatility and low toxicity, with potential for development into aging asphalt binder rejuvenators [18]. Xiong et al. came up with a review of biochar based catalysts for chemical synthesis, biofuel production, and pollution control [19]. Bio-oil generated through a fast pyrolysis, cooling, and condensation process has a similar chemical composition to petroleum asphalt binders and a higher amount of maltene [20]. Chen et al. [3] chose three vegetable oil samples with similar physical properties to their virgin binders to prove bio-oil can effectively soften aged asphalt. The asphaltenes contents of aged asphalt were decreased after adding bio-oil, based on laboratory simulation. Therefore, bio-oil meets the basic requirements of an asphalt binder rejuvenator, as it softens aged asphalt binder. Raouf [21] studied the physical and chemical properties of three different types of bio-oils (namely oak, switchgrass, and corn straw) as bio-asphalt binders. It was found that the logarithmic linear relationship between viscosity, temperature, and shear rate of the oak-based bio asphalt binder was similar to that of the petroleum asphalt binder. However, the bio-binder was found to be more sensitive to temperature than the traditional petroleum asphalt binder.

Fini et al. [22,23] studied the characteristics of a bio-binder generated from swine manure. The results show that whilst the addition of a bio-binder to an asphalt binder potentially improves the low-temperature properties and workability of a petroleum asphalt binder, it inversely reduces the high-temperature properties. Additionally, You et al. [24] also found that a bio-binder made of swine manure can potentially be used as an asphalt binder modifier to reduce the cracking temperature of PG 64–22 asphalt binders by about 4.2~4.6 °C, with the addition of 10% bio-binder (by weight of the asphalt binder). In their study, Zofka and Yut [25] extracted bio-oils from waste coffee grounds and observed that the bio-oils did not change the temperature susceptibility of the base asphalt binder, nor did they serve as antioxidants in the asphalt binder. However, Jalkh et al. [26] observed that blending the extracted bio-oil with an asphalt binder recovers the linear behavior of the asphalt binder, increases the softening process of the blend matrix, and lowers its susceptibility to permanent damage at low stress levels. On the other hand, Wen et al. [27] reported that the addition of waste cooking oil decreases resistance to fatigue and rutting.

Chen [28] investigated the influence of waste cooking oil and cotton seed oil on the high-temperature performance of rejuvenated asphalt binders using a dynamic shear rheometer (DSR) test device. Likewise, Tang [29] used three bio-oils derived from corn stover, oak wood, and switch grass to comparatively evaluate their susceptibility to oxidative aging, and their ability to rejuvenate aged asphalt binders. From the study findings [28,29], it was concluded that bio-oils had the potential to minimize oxidative aging and rejuvenate aged asphalt binder.

As reported in the literature, some studies have also explored the use of rejuvenators or modifiers derived from soybean oil, denoted herein as SBO. Seidel [30] conducted high-temperature rheological tests of asphalt binders modified with soy fatty acids (SFAs), and found that small proportional additions of SFAs can produce asphalt binders that are less stiff and more workable. Elkashef et al. [31,32,33,34] studied the physical and chemical properties of an aged asphalt binder that was rejuvenated with SBO using the DSR, BBR, FTIR and GC-MS tests, respectively. The test results show that the SBO rejuvenator can significantly improve the low-temperature and fatigue performance of an asphalt binder, including reducing its sensitivity to temperature.

To define the interfacial reaction between the rejuvenator and the asphalt binder, research has been dedicated to studying the interfacial reaction between a rejuvenator and an aged asphalt binder. The diffusion mechanism between the rejuvenator and the aged asphalt binder is considered to be one of the most technical concerns, with respect to the rejuvenation mechanisms and the use of bio-oil rejuvenators such as SBO [35,36]. Karlsson [37] evaluated and successfully modeled the internal diffusion behavior of microencapsulated rejuvenators in aged asphalt binders using the FTIR-ATR tests. Ding [38] employed molecular dynamic (MD) simulations to explore the diffusion between original and aged asphalt binders. Girimath et al. [39] tested bio-oil consisting of aromatic chemical compounds and oxygen-containing compounds, which have the beneficial ability to improve the properties of asphalt binders. Yang et al. [40] tested bio-binders using gel permeation chromatography (GPC) and found that the components of bio-oil are similar to those of asphalt binders. The MD simulation results were successfully verified using GPC (gel permeation chromatography), with the corresponding results indicating that the diffusion of large molecules in the asphalt binder is a critical factor in the diffusion of asphalt binders.

Xiao [41], Zadshir [42], and Xu [43] used the concept of the diffusion coefficient with MD simulations to study the diffusion behavior of rejuvenators and asphalt binders. Laboratory experimentations (such as the DSR, FTIR, etc.) were also performed to verify the MD simulation results. The study results indicated that MD simulations, along with laboratory testing, were able to successfully model and quantify the rejuvenator diffusion behavior, along with its impact on the molecular structure and thermodynamic properties of the asphalt binders in RAP materials.

To improve on the utilization ratio of RAP, and understand the diffusion and rheological properties of bio-oil-rejuvenated aged asphalt (BRAA), the molecular structure of bio-oil was measured using an element analyzer (EA), gel permeation chromatography (GPC), and Fourier infrared spectrometer (FTIR) in this study. Molecular models of BRAA were built and the diffusion parameters were determined using MD simulations. In the study, the rheological properties of BRAA were measured and quantified using a DSR test device.

## 2. Study Work Plan and Materials Used

The research methodology and study work plan incorporated the following key activities: (a) material procurement; (b) aging of the asphalt binders and sample preparation; (c) laboratory experimentation and testing; (d) molecular dynamic simulations; and (e) data analysis and synthesis to draw conclusions and recommendations. The materials and sample preparation are discussed below, followed by the rest of the work activities in subsequent sections.

### 2.1. Base Asphalt Binder

The base asphalt binder used in this study was A-70# petroleum asphalt binder procured locally in China. The basic properties of the asphalt binder were evaluated based on the Chinese specification JTG E20-2011. The physical properties and technical indices of the base asphalt binder are listed in Table 1.

### 2.2. The Aged Asphalt Binder

In accordance with the AASHTO T 240 13-5 [44] requirements, the A-70# petroleum asphalt binder was subjected to short-term aging using the rolling film oven test. A 350 g sample of the asphalt binder was placed in a glass bottle, 140 mm in height and 64 mm in diameter. The glass bottle was then inserted into a rotary oven at 163 °C and rotated at 15 r/min for 85 min. A hot air flow was continuously administered at 4000 mL/min.

The pressure-aging vessel (PAV) was then employed to simulate long-term aging based on the ASTM D454 13-6, ASTM D 6521 13-7 [45], and ASTM D572 13-8 standard test methods. The sample plates with 50 ± 0.5 g of the asphalt binder that had been exposed to the rolling thin film oven test (RTFOT) were placed in the vessel for aging under a specified pressure of 2070 kPa and at 100 °C for 20 h. The physical properties and technical indices of the PAV-aged asphalt binder, measured based on the Chinese specification JTG E20-2011, are listed in Table 2.

### 2.3. The Soybean Oil Rejuvenator

In this study, the bio-oil generated from soybean oil and blended with some stable components was used as the rejuvenator. Soybean oil is a sepia (reddish-brown color) viscous liquid at room temperature. The density of the bio-oil was close to that of petroleum asphalt binder. The physical properties of the soybean oil, measured using the Chinese specification JTG E20-2011, are listed in Table 3.

### 2.4. BRAA Sample Preparation and Bio-Oil Dosages

About 500 g of the aged asphalt binder was placed into a stainless-steel asphalt binder extractor and heated to a temperature less than 110 °C. Thereafter, 1.0%, 2.0%, 3.0%, 4.0% and 5.0% of the residual soybean oil by weight (wt) of the asphalt binder was added, respectively. The residual soybean oil was then sheared for 1.5 h using a high-speed shear device, until the residual soybean oil was completely dissolved in the hot aged asphalt binder. Five BRAA sample replicates were prepared per rejuvenation dosage.

## 3. Laboratory Testing and Simulations

Laboratory experimentations were conducted to measure and characterize the morphological structure and rheological properties of BRAA. These laboratory tests are discussed in this section and include the FTIR, GPC, EA, and DSR, respectively. MD simulations were conducted to quantify the diffusion parameters of the BRAA molecules, and are also discussed in this section. For each respective laboratory test and MD simulation, a minimum of three samples/specimens or test replicates was used per asphalt binder and BRAA per soybean oil dosage under each test condition.

### 3.1. The Fourier Infrared (FTIR) Spectrometer Test

Infrared spectroscopy is one of the common methods used for the structural analysis and identification of compounds. It can record changes in the transmission light intensity in the corresponding absorption region of the molecules in the compound, which occur when a beam of infrared light with a continuous frequency variation is used to irradiate the compound. This is due to the molecules in the compound absorbing the radiation of some wavelengths of the infrared light, leading to the transition of the molecular vibration energy level and rotation energy level. Even though the vibrational forms of atoms are complicated, the infrared spectra of different substances have their own characteristics. Therefore, the chemical structure of the substance can be identified through the shape, position, and intensity of the band in the infrared spectrum. Additionally, the content of the corresponding components can be identified through the intensity of the characteristic absorption peak.

For FTIR-ATR testing [46], a Bruker Tensor 37 with an attenuated total reflectance device was employed with a resolution of 4 cm^−1^, scan period of 32 times, and scan range of 4000~400 cm^−1^. The ATR crystal was made of zinc selenide.

### 3.2. The Gel Permeation Chromatography (GPC) Test

The GPC test is a method that uses a column composed of special porous fillers to separate a polymer solution according to the molecular size in the column. The GPC test was adopted in this study to analyze the molecular weight distribution characteristics of the virgin asphalt binder, the aged asphalt binder, and the asphalt binder rejuvenated with bio-oil. When the polymer solution enters the column, solute molecules try to penetrate the internal pores of the packing because of the difference in the concentrations. The smaller molecules in the sample can enter all the pores, but the larger molecules in the sample can only diffuse into the larger holes, and flow through the packing particle gap if they cannot flow into the holes directly. Under the solvent leaching process, the largest molecules in the sample will be the first to flow out of the column, followed by the smaller molecules. The smallest molecules will remain and be the last to flow out, thus achieving the purpose of separating the sample according to the sizes of the molecules.

The molecular weight distribution curves of the asphalt binders and SBO were obtained by employing a 515–410 gel permeation chromatograph test. The solvent of the carrier was tetrahydrofuran, which has good solubility with the asphalt binder. During testing, the temperature of the columns was kept at 40 °C with a mobile phase flow rate of 1 mL/min. The molecular precipitation and termination times in the separation spectrum were divided into 13 equal parts, namely, 1 to 13. These parts, namely, 1–5, 6–9, and 10–13, were defined as the spectra of the large molecular size (LMS), medium molecular size (MMS), and small molecular size (SMS), respectively.

### 3.3. The Elemental Analyzer (EA) Test

In this study, the element analysis of the BRAA was conducted using the elemental analyzer Vario EL Ⅲ [47]. The measurement of asphalt binder carbon (C), hydrogen (H), nitrogen (N), and sulfur (S) contents, for the quantitative analysis of the content of oxygen (O), was accomplished using the differential method. The CHNS mode was used for testing with benzene sulfonic acid as the standard solvent.

### 3.4. The Dynamic Shear Rheometer (DSR) Test

In this study, the DSR test was conducted to measure and quantify the rheological properties of the modified asphalt binder, namely, BRAA. The tests were performed in accordance with the ASTM D7175-15 13-11 [48] standard test method. The DSR test temperature ranged from 0 to 88 °C, in 6 °C increments, at a constant frequency of 10 rad/s [49,50,51]. A parallel plate geometry, comprising of a 25 mm diameter with a 1 mm gap (high temperature) and an 8 mm diameter with a 2 mm gap (low temperature), was used for holding the asphalt binder specimens during the rotating shear testing.

As a viscoelastic material, the viscoelastic properties of the asphalt binder and the BRAA under different temperature conditions were quantified using the complex shear modulus (G*) and phase angle (δ), respectively [52]. In line with the Superpave asphalt binder’s specification [53], the G*/Sin (*δ*) parameters for the original (unaged virgin) asphalt binder and the short-term-aged asphalt binder were limited to 1.0 kPa and 2.2 kPa, respectively [54].

### 3.5. Molecular Dynamic (MD) Simulations

Asphalt binder is composed of four key components, namely, saturates (S), asphaltenes (A), resins (R), and aromatics (A), often denoted as SARA [55]. Due to the influence of high-temperature oxidation and given the assumptions (i.e., mass ratios) made in this study, the main change seen in the asphalt binder’s components throughout the process of aging is that the resins (R), which are the lighter components, are converted into asphaltenes (A). Macroscopically speaking, this makes the asphalt binder decrease in plasticity and an increase in brittleness, while the contents of the saturates (S) and aromatics (A) components did not change significantly. However, these should not be taken as the exclusive aging-related trends, as the response (aging) trend may vary depending on the assumptions made, such as the SARA fractions, etc.

For this study, the mass ratios of the four components in the unaged asphalt binder were taken as follows: m (asphaltenes (A)): m (saturates (S)): m (aromatics (A)): m (resins (R)) = 1.0, 2.7, 3.1, and 3.9, respectively. In the aged asphalt binder, the ratios were 1.0:1.0:1.0:1.1, respectively. That is, while the fractional mass ratio of the asphaltenes remained the same at 1.0, the rest of the components decreased with aging, i.e., from 2.7 to 1.0 for saturates, 3.1 to 1.0 for aromatics, and 3.9 to 1.1 resins. Thereafter, the mass ratios of the four components were converted into molar ratios for the SARA test.

**Figure 1:**
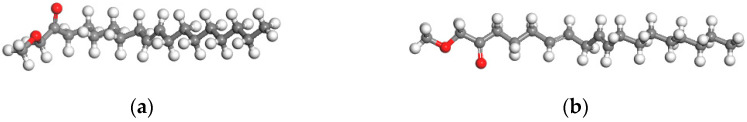
Bio-oil model: (**a**) C_18_H_32_O_2_ and (**b**) C_18_H_34_O_2_.

In this study, the Amorphous Cell module in the Materials Studio software was used for molecular modeling and dynamic simulations. For the simplicity of the MD simulations, the molecular structure of the bio-oil was replaced with the main molecules shown in Figure 1.

**Figure 2:**
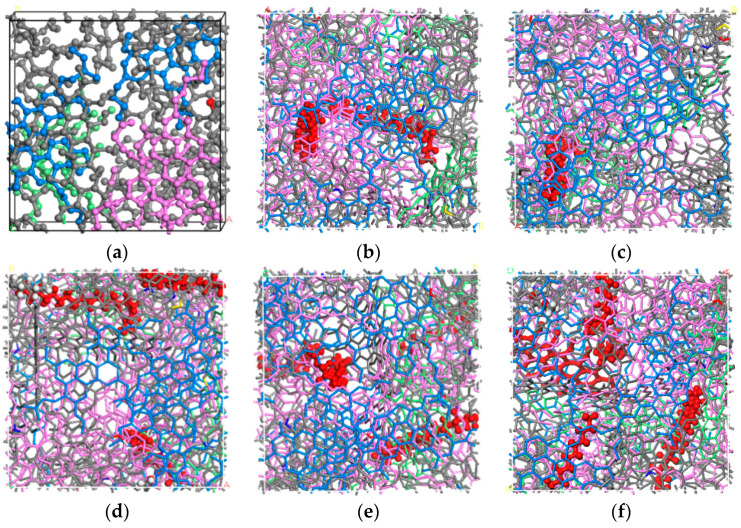

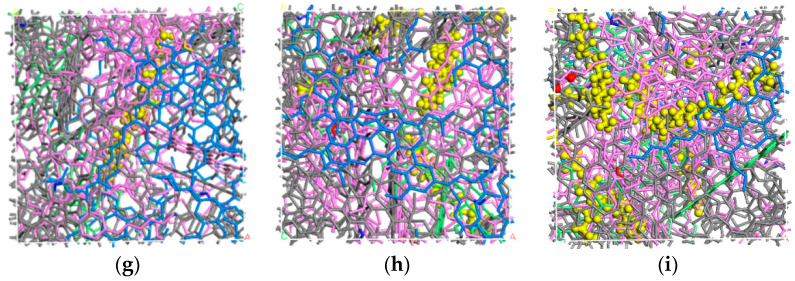
(**a**) A-70# base asphalt binder; (**b**–**f**) 1~5% C_18_H_32_O_2_, red components; and (**g**–**i**) 1~3% C_18_H_34_O_2_, yellow components.

Both the virgin unaged and aged asphalt binders used the four-components model, namely, SARA. The molecular models of BRAA were built according to the detailed contents of all the components, consisting of both the bio-oil (soybean oil) and the aged asphalt binder. The simulated molecular models of BRAA are shown in Figure 2. In Figure 2, the color-coding scheme is as follows: blue = aromatics, gray = asphaltenes, green = resins, pink = saturates, red = 1−5% C_18_H_32_O_2_, and yellow = 1−3% C_18_H_34_O_2_.

Some basic parameters determined using MD simulations included the density, solubility parameter, diffusion coefficient, radius of gyration, viscosity, and bulk modulus. Solubility parameters indicate the degree of compatibility among substances, and also reflect the degree of stability in the blended matrix system. Based on the principle of similar solubility parameters, it is possible that there is better compatibility among the substances in the system.

When the maximum difference of the solubility parameters is less than 4.1 (J/cm^3^)^1/2^, there may be better compatibility between the substances. In this study, the solubility parameters expressed in Equation (1) were used to model and verify the stability of the asphalt binder matrix system [56]:

(1) $$\delta =\sqrt{CED}=\sqrt{\frac{e}{v}}$$

In Equation (1), *δ* is the solubility parameter, *CED* is the cohesive energy density, *e* is the cohesive energy, and *v* is the volume of the model.

Diffusion is defined as the movement of molecules from high-concentration regions to low-concentration regions. Due to the complex nature of the asphalt binder materials, diffusion in asphalt binder is a complex phenomenon. Precise prediction and control of the rejuvenators′ diffusion into the RAP asphalt binder is still a challenge. Generally, Fick′s Law is used to mathematically describe diffusion phenomenon, as shown in Equation (2) [57]:

(2) $$\frac{\partial c}{\partial t}=D\times \frac{\partial^{2}c}{\partial x^{2}}$$

where *c* is the concentration, *t* indicates the time, *x* refers to the position, and *D* is the diffusion coefficient. The diffusion coefficient is typically used to represent the diffusion between the asphalt binder and rejuvenator, as shown in Equation (3) [38]:

(3) $$D=\frac{a}{6}$$

where *D* is the diffusion coefficient, and *a* is the slope of the line plot of the mean square displacement (MSD) versus simulation time.

MSD is defined as the deviation in a particle’s position towards a referenced position after free diffusion in the system at a certain time, and is mathematically represented by Equation (4) [58]:

(4) $$MSD=\frac{1}{N}\sum_{i=1}^{N}{\left[r_{i}(t)-r_{i}(0)\right]}^{2}$$

where *t* is the free-diffusing time of the atoms in the system, *MSD* is the mean square displacement, *N* is the number of moving atoms in the system, *r_i_*(*t*) is the position vector of atom *i* at certain time *t*, and *r_i_*(0) is the initial position vector of an atom *i*.

Asphalt binder molecules usually have a high amount of spatial conformations, and the radius of gyration (*R*_g_) can be used to describe the spatial conformations according to some characteristic properties, as expressed in Equation (5) [59]:

(5) $$R_{g}=\sqrt{\left(\sum \frac{\left(x_{i}^{2}+y_{i}^{2}+z_{i}^{2}\right)}{N}\right)}$$

In Equation (5), *x*, *y*, and *z* are the distances between all atoms at different coordinates.

Viscosity is one of the most indicative parameters used for modeling and estimating asphalt binder performance. This is because viscosity is closely related to the mixing and compaction temperatures used in the construction of hot mix asphalt (HMA). In this study, the viscosity of the constructed asphalt binder molecular models was calculated for comparison with the actual asphalt binder viscosity to verify the accuracy of the constructed model. Lawes’ model, shown in Equation (6), was used to determine the viscosity of the simulated asphalt binder models:

(6) $$\eta_{D}=\frac{\rho RTR_{g}^{2}}{6MD}$$

where *η_D_* is the viscosity, *D* is the diffusion coefficient, *ρ* is the density of the system, *R* indicates the molar gas constant, *T* is the temperature, *R*_g_ is the radius of gyration, and *M* is the molecular weight.

Bulk modulus can be determined based on MD simulations using the dynamical stepwise compression process at multiple temperatures via Equation (7) [60]:

(7) $$B=-V_{sp}{\left(dV_{sp}/dP\right)}_{T}^{-1}=-{\left(d(\ln V_{sp})/dP\right)}^{-1}$$

In Equation (7), *B* is the bulk modulus (inverse of the isothermal compressibility), *V_sp_* is the specific volume, and *P* is pressure.

## 4. Results, Analysis, and Discussions

The results for the asphalt binder, bio-oil (rejuvenator), and BRAA, derived from both MD simulations and laboratory experimentations, are presented, analyzed, and discussed in this section of the paper. These results relate to the elemental analysis, the molecular weight distribution of the bio-oil, the BRAA functional groups, the density, the diffusion coefficient, the viscosity, the bulk modulus, the solubility parameters, the radius of gyration, and the rheological properties, namely, the complex shear modulus and phase angle. Note that this study investigated the anti-aging effects of the rejuvenator as well as the effects of the rejuvenator dosage, in comparison to both base (unaged) and aged asphalt binders. Therefore, the results presented herein include comparisons with both base (unaged) and aged asphalt binders.

### 4.1. EA Test Results and Compositional Analysis

As shown in Table 4, the molecular formula for bio-oil is either C_18_H_32_O_2_ or C_18_H_34_O_2_, according to the C/H/O ratio. Compared with the data for the base asphalt binder, the C contents of BRAA were quantitatively lower than in the base asphalt binder, and vice versa for the O contents. Ultimately, these results indicate the superior anti-thermal oxidative aging resistance of BRAA, suggesting that bio-oil could potentially improve the anti-thermal oxidative aging resistance of aged asphalt binders.

In general, the asphalt binder creates a S=O chain during oxidative aging. This phenomenon (S=O chain generation) suggests that the S components/contents can easily oxidize and age. However, Table 4 shows that the S contents of BRAA were lower than in the base asphalt binder, suggesting that BRAA does not oxidatively age easily.

As reported in the literature, previous studies have observed that the VOC (especially PAHs) of the asphalt binder is mainly derived from its N organic components. By comparison, Table 4 shows that the N contents of BRAA were quantitatively lower than in the base asphalt binder. These results (Table 4) suggest that BRAA could potentially produce lower VOC emissions than the base asphalt binder during the heating, mixing, and/or paving processes. This ultimately implies that BRAA is an environmentally friendly road material.

### 4.2. GPC Molecular Weight Distribution Results for the Bio-Oil

**Figure 3:**
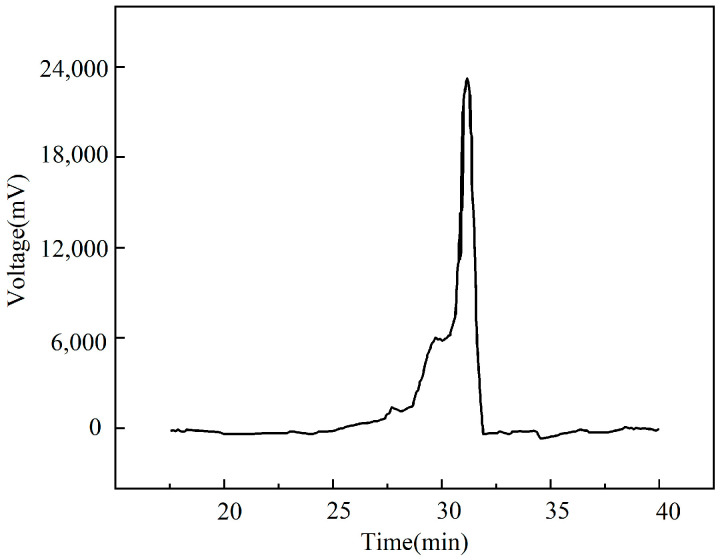
Molecular weight distribution of the bio-oil.

Mw is average weight, Mn is average number, and Mw/Mn is molecular weight distribution. As shown in Figure 3, the voltage of bio-oil ranged from 30.25 to 32.50 min in the GPC test. In line with the EA test results, the molecular weights corresponding to these voltage readings ranged from 280 to 282 g/mol. The average weight, relative to the molecular mass, is the average of the simple molecular weight, reflecting the existence of macromolecules. The average number, relative to the molecular mass, is derived from the number of existing molecules. Hence, the molecular weights of C_18_H_32_O_2_ and C_18_H_34_O_2_ are 280 and 282 g/mol, respectively.

### 4.3. FTIR Compositional Analysis and Functional Group Results

**Figure 4:**
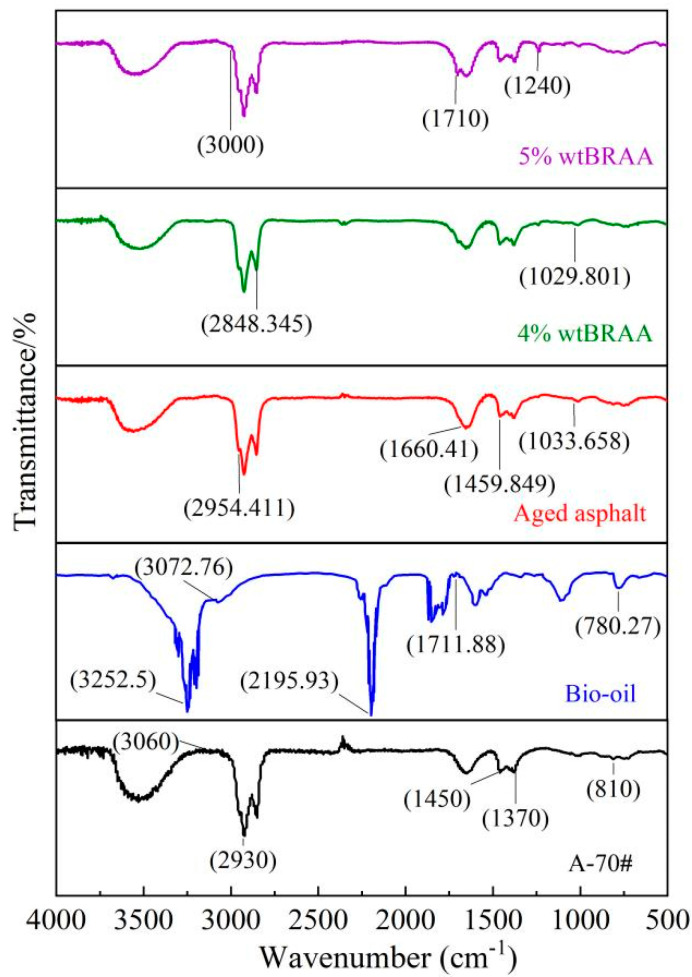
FTIR spectroscopy of the soybean oil.

The chemical composition and functional groups of the A-70# base asphalt binder, soybean oil, aged asphalt binder, BRAA with 4.0% bio-oil, and BRAA with 5.0% bio-oil were analyzed using an FTIR spectrometer. The FTIR analysis results are shown in Figure 4.

From the FTIR spectra of the base asphalt binder in Figure 4, it can be seen that the A-70# virgin asphalt binder had significant absorption peaks at the same absorption wavenumbers (2930 cm^−1^, 3060 cm^−1^, 1450 cm^−1^, and 1370 cm^−1^, respectively) as the aged asphalt binder. The base asphalt binder was mainly composed of saturated hydrocarbons, aromatic compounds, aliphatic amines, carboxylic acids, and heteroatom derivatives. The strong peak at 2930 cm^−1^ was ascribed to the methylene C-H bond stretching vibration. The two strong absorption peaks at 1450 cm^−1^ were attributed to the flexural vibrations of the saturated C-H bond.

The FTIR spectra of the bio-oil in Figure 4 indicate remarkable absorption peaks near 3300 cm^−1^ and 3252 cm^−1^, caused by the alcohol or aldehyde O-H bond stretching vibration, while the expansion vibration of the aromatic C-H bond appeared at 3072 cm^−1^. The absorption peak at 1711 cm^−1^ was attributed to the expansion vibration of C=O, which is one of the characteristic absorption peaks of vegetable-based oil. The absorption peak at 780 cm^−1^ represents the carbon chain skeleton vibration peak.

For the aged asphalt binder, the carbonyl stretching vibration absorption peak appeared in the infrared spectrum within the range of 1650~1900 cm^−1^ [61] (here, 1660 cm^−1^ is shown in Figure 4), which was rarely disturbed by the other groups. In contrast to the aged asphalt binder, the two peaks were at 1710 cm^−1^ and 1033 cm^−1^ for BRAA with the addition of 5.0 wt. % bio-oil. This is because the addition of the bio-oil rejuvenator increased the content of maltene, with a lighter molecular weight, in the asphalt binder matrix, and softened the aged asphalt binder. For future follow-up studies and to further supplement these results/findings, the use of FTIR indices should be explored as a quantitative measure of absorption and an indicator of the rejuvenation effects (i.e., at specific wavenumbers).

### 4.4. Density Results and Synthesis

**Figure 5:**
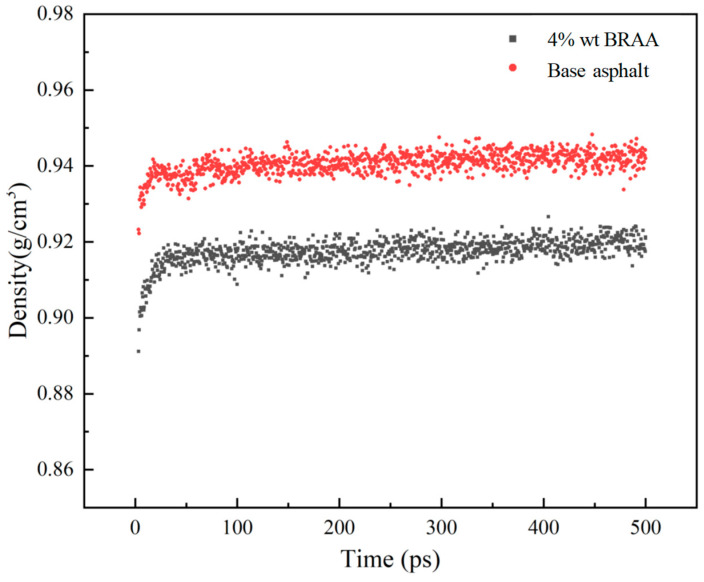
The density curves of asphalt binders at 298.15 K.

The density curves of the base asphalt binder and BRAA with 4 wt. % bio-oil additive, respectively, are shown in Figure 5. As shown in the figure, the density of BRAA is quantitatively lower than that of the base asphalt binder. This suggests that the bio-oil could potentially reduce the density of the aged asphalt binder and make it lighter.

### 4.5. Diffusion Coefficient Results from MD Simulations

As shown in Table 5, the diffusion coefficient of BRAA obtained from the MD simulations shows that the D_Bio-oil_ was the highest, followed consecutively by D_Resins_, D_Aromatics_, D_Asphaltenes_, and D_Saturates_, respectively. These results show that the bio-oil could potentially improve diffusion in the aged asphalt binder, and promote the regeneration of the aged asphalt binder. The diffusion of resins and asphaltenes in the matrix system is very important. Accordingly, the diffusion coefficients of resins and asphaltenes decreased with an increase in the bio-oil content to 4.0 wt. %. Thus, the level of bio-oil content could potentially lower the diffusion coefficients of the BRAA components and inhibit the net diffusion of some of these components.

In general, the diffusion response behavior of BRAA at different temperatures was different at different bio-oil contents. As shown in Table 6, the diffusion coefficients of the BRAA components generally increased with the temperature change from 0 °C (273 K) to 25 °C (298 K) for saturates and resins, and from 0 °C (273 K) to 45 °C (318 K) for asphaltenes, aromatics, and bio-oil. At 45 °C (318 K) and above, a general decline is noted, followed by an unexplained increase at 80 °C (353 K). For all the components, the peaks seem to occur at either 25 °C (298 K) or 45 °C (318 K), while the lowest diffusion coefficient was registered at 60 °C (333 K) for each component element. Evidently, these results show that the BRAA components’ diffusion and the regeneration of the aged asphalt binder could potentially be improved at a certain temperature. For this study, 25~45 °C (298~318 K) was determined to be the optimum temperature range for maximizing the diffusion coefficients.

In the matrix system shown in Table 6, the D_Bio-oil_ generally exhibited the highest magnitude in terms of the diffusion coefficient, followed by D_Aromatics_, D_Resins_, D_Asphaltenes_, and D_Saturates_ (smallest), respectively. In other words, the diffusion coefficient varied as a function of the bio-oil (soybean-derived) dosage. For the bio-oil contents exemplified in Table 6, the peak diffusion occurred within the optimum temperature range of 25~45 °C (298~318 K). However, the observed reduction above this temperature range, followed by the unexplained increase in the magnitude of the diffusion coefficient at 80 °C (353 K), warrants further investigations in future follow-up studies.

### 4.6. Viscosity and Bulk Modulus Results from MD Simulations

**Figure 6:**
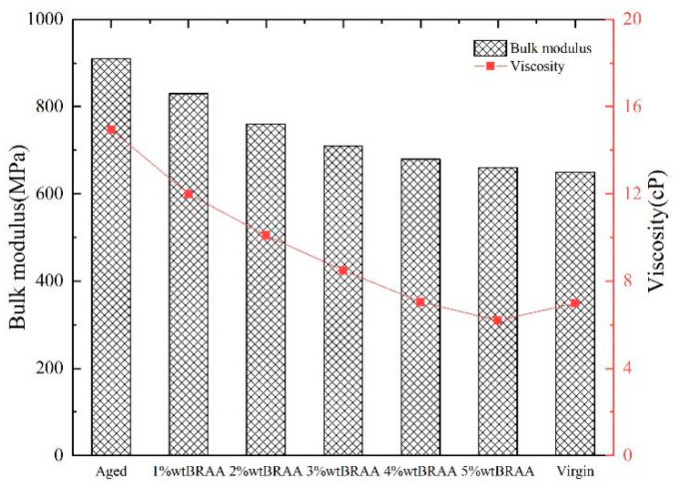
BRAA viscosity and bulk modulus results at 298.15 K.

As shown in Figure 6, the viscosity and bulk modulus in the MD simulations exhibited a similar declining response trend with an increase in the bio-oil content. That is, the magnitude of both the viscosity and bulk modulus decreased progressively with the addition of the bio-oil from 1% (highest value) to 5% (lowest value). By comparison, however, Figure 6 shows that the values for BRAA were quantitatively lower than those for the aged asphalt binder, but were relatively higher than the unaged virgin base asphalt binder. This means that the bio-oil (soybean-derived) rejuvenator has the potential to reduce the viscosity and bulk modulus of the aged asphalt binder, and the greater the bio-oil dosage, the greater the reduction, particularly for the viscosity parameter. Overall, these results indicate good prospects when using a soybean-oil derived rejuvenator to rejuvenate aged asphalt binders and to enhance viscoelastic properties.

As theoretically expected, Figure 6 shows that both the viscosity and bulk modulus of the aged asphalt binder were quantitatively higher than those of the unaged virgin base asphalt binder. On the other hand, the values for BRAA rejuvenated with bio-oil additive fall in between those of the aged and the unaged asphalt binders. These observations suggest that the bio-oil rejuvenator, which is soybean-derived, has the potential to optimize the viscosity and bulk modulus below that of the aged asphalt binder and above that of the unaged virgin asphalt binder, particularly for dosages less than 5%. On this basis, it can also be inferred from Figure 6 that the optimum dosage of bio-oil rejuvenator should not exceed a 4% content of soybean oil.

### 4.7. Solubility Parameter Results from MD Simulations

As shown in Table 7, the solubility parameters determined from MD simulations indicate that the solubility of the bio-oil was comparable to that of the asphaltenes, aromatics, and aged asphalt binder. That is, the closer the values are in magnitude, the greater the solubility of those specific material components within each other. Thus, the results in Table 7 indicate that the bio-oil, which is soybean-derived, could easily dissolve into asphaltenes, aromatics, and the aged asphalt binder. Ultimately, this indicates the potential for the bio-oil to chemically react with and rejuvenate the aged asphalt binder.

The ΔS* values in Table 7 are all less than 2.000 in magnitude. This means that the bio-oil could dissolve into the aged asphalt binder and the BRAA components, a characteristic property that is critical when regenerating aged asphalt binder. In Table 7, the order of ΔS* magnitude is as follows: bio-oil–saturates > bio-oil–aged asphalt binder > bio-oil–asphalt binder > bio-oil–asphaltenes > bio-oil–resins > bio-oil–aromatics. In terms of solubility ranking, this means the bio-oil is more soluble in the aromatic’s components, and the least in the saturates.

### 4.8. Radius of Gyration Results from MD Simulations

**Figure 7:**
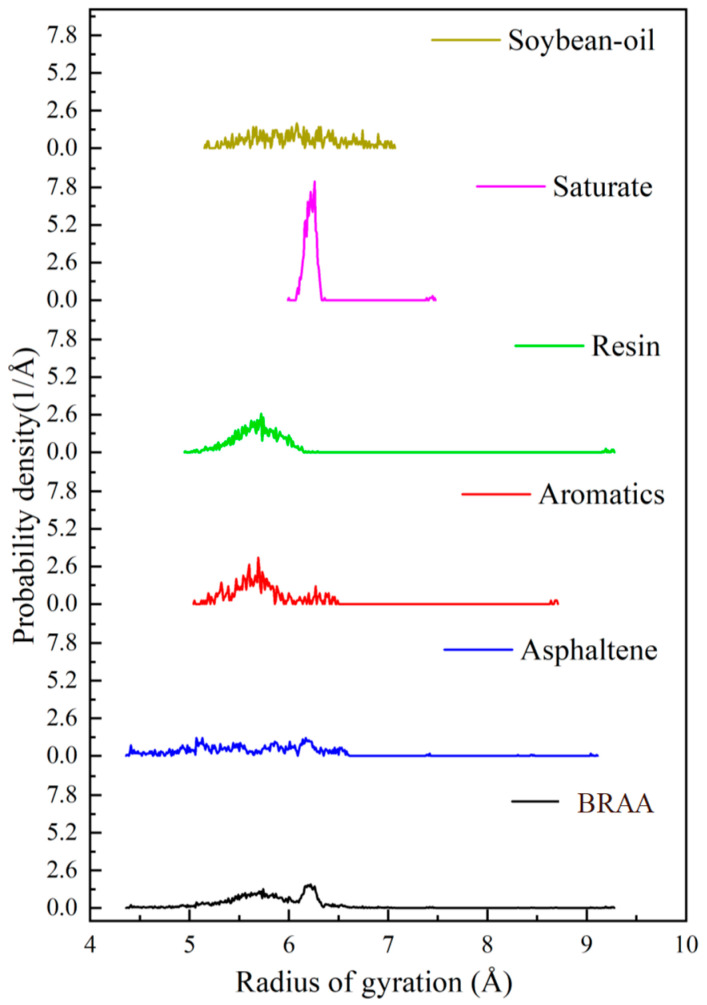
Radius of gyration results.

As shown in Figure 7, the radii of gyration of the four components, namely, SARA, are different. The rank order of reduction in the radius of gyration was saturates, aromatics, resins, bio-oil and asphaltenes, which gives rise to different molecular structures. The addition of bio-oil has the potential to alter the molecular structure of the aged asphalt binder and enhance its regeneration capability. The probability densities of all the components, i.e., saturates, aromatics, resins, bio-oil, and asphaltenes, were 8.1, 3.9, 2.6, 1.3, and 0.9–1.1 Å, respectively, thereby indicating that the radius of gyration of saturates is superior. In Figure 7, the rejuvenated profile of BRAA (with 4.0% bio-oil) indicates that the diffusion of the bio-oil would potentially promote and enhance molecular movement within the aged asphalt binder. Additionally, the bio-oil would also help to supplement some components of the aromatics and/or saturates in the aged asphalt binder, and allow the aged asphalt binder to form a new stable matrix system.

### 4.9. DSR Rheological Results and Synthesis

**Figure 8:**
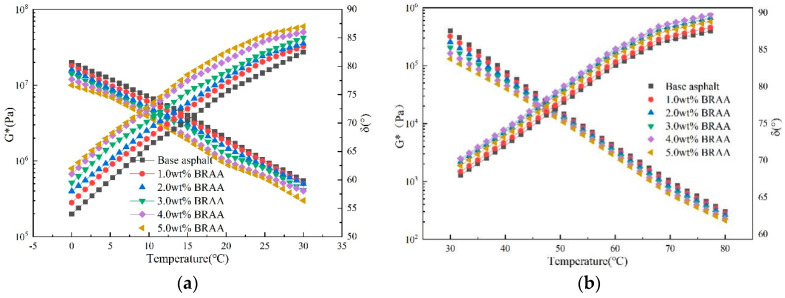
DSR test results: (**a**) low temperature = 0–30 °C, (**b**) high temperature = 30–80 °C.

In both low and high DSR temperature testing, Figure 8 shows that the complex shear modulus (G*) exhibited a decreasing response trend with increases in the test temperature and bio-oil dosage. However, the opposite response trend (i.e., increasing) can be observed to be true for the phase angle (*δ*). When looking at the BRAA results in Figure 8, there is an inherent implication that bio-oil, within a certain dosage range, could potentially improve the low-temperature and high-temperature properties of the aged asphalt binder. Furthermore, the results also indicate that the bio-oil exhibited the potential of not only enhancing the low-temperature properties (i.e., anti-cracking) of aged asphalt binder, but also improving its high-temperature anti-aging properties, particularly with BRAA dosages less than 4.0%wt.

In Figure 8, it is apparent that the 4.0 wt. % and 5.0 wt. % BRAAs are fairly distant from the base asphalt binder’s response curves. For the high-test temperature regime in Figure 8b, this means that the excessively low G* and high δ values compared to the base asphalt binder, such as 4.0 wt. % and higher BRAA, may indicate a potential susceptibility to high temperature rutting problems. Therefore, the optimum bio-oil dosage should not exceed 4.0 wt. %. Considering that lower G* and higher *δ* values compared to the base asphalt binder are desired to mitigate low-temperature cracking, it is apparent from Figure 8a that any bio-oil dosage greater than 1.0 wt. % would suffice, i.e., 1.0~4.0 wt. %. On this basis, and considering the viscosity and bulk modulus results in Figure 6, 3.0 wt. % could be selected as the optimum bio-oil (soybean) dosage.

### 4.10. Data Quality and Descriptive Statistical Analysis

As previously mentioned in Section 2, the test results represent an average of five BRAA sample replicates per rejuvenation dosage per test condition. Descriptive statistical analyses in terms of the coefficient of variation (COV) were carried out to comparatively evaluate the test data quality and consistency in this study [51,59,60]. Quantitatively, the lower the COV value, the better the consistency and data quality [61]. In general, the obtained COV values, which ranged from 0.09% to 11.05%, exhibited reasonably acceptable test repeatability, data quality, and consistency [51,59,60,61]. Given the materials evaluated and the laboratory test conditions considered, this good repeatability and relatively low variability in the test data (i.e., COV < 20%) can be partly attributed to good workmanship, proper machine calibration, the use of trained operators, etc.

## 5. Conclusions and Recommendations

In this study, the diffusion and rheological properties of soybean bio-oil used to rejuvenate aged asphalt binder (BRAA) were investigated using molecular dynamic simulations and laboratory experimentations. From the results and findings of the study, the following conclusions and recommendations were drawn:

- BRAA exhibited lower VOC emissions and more environmental friendliness than the base asphalt binder;
- The molecular weight of the bio-oil, which was soybean-derived, was found to be 280~282 g/mol, and could be represented using either the C_18_H_32_O_2_ or the C_18_H_34_O_2_ chemical formula. For the materials evaluated, the bio-oil indicated the potential ability to enhance the diffusion and promote the regeneration of the aged asphalt binder;
- The bio-oil evaluated, which was soybean-derived, exhibited solubility in the aged asphalt binder, with potential to improve its viscoelastic properties and the diffusion, and to promote its regeneration. For a bio-oil dosage of 4.0%, the diffusion coefficients of the BRAA components were 1.52 × 10^−8^, 1.33 × 10^−8^, 3.47 × 10^−8^, 4.82 × 10^−8^ and 3.92 × 10^−8^, respectively;
- The regeneration mechanism of BRAA indicates that the chemical diffusion of the bio-oil enhanced the molecular movement within the aged asphalt binder, including supplementing the aromatics and saturates components to form a new stable asphalt binder matrix system.

Overall, the study has indicated that bio-oil derived from soybean is a promising regeneration agent for rejuvenating aged asphalt binders, with 4.0 wt. % as the tentative optimum dosage. However, whilst the study results are plausible, experimentation with different base asphalt binders and other laboratory tests, such as cracking and moisture evaluation, along with field validation, is warranted in future studies. To further supplement the results/findings presented herein, future studies should also explore the use of FTIR indices as a quantitative measure of absorption and an indicator of the rejuvenation effects. Nonetheless, the study contributes to the state-of-the-art by supplementing and enriching the existing/current literature through the provision of a datum reference for using soybean-derived oil as a potential regenerant agent to rejuvenate aged asphalt binders.

## Figures and Tables

**Table 1:** Technical indices of the A-70# petroleum asphalt binder.

Technical Index		Unit	Specification	Test Result
Penetration (25 °C, 100 g, 5 s)		0.1 mm	60~80	72
Penetration index, PI		-	−1.5~+1.0	−1.45
Softening point, T_R&B_		°C	≥46	46.2
Ductility (15 °C, 5 cm/min)		cm	≥100	138
Ductility (10 °C, 5 cm/min)		cm	≥15	22.51
Density @15 °C		g/cm^3^	/	1.043
Wax content		%	<2.2	2.18
Dynamic viscosity @60 °C		Pa∙s	≥180	248
Kinematic viscosity @135 °C		Pa∙s	/	0.485
After RTFOT (163 °C, 85 min)	Mass change	%	−0.8~+0.8	0.05
	Penetration ratio @25 °C	%	≥61	73.85
	Ductility (10 °C, 5 cm/min)	cm	≥6	9

Legend: RTFOT = rolling thin film oven test.

**Table 2:** Technical indices of PAV-aged asphalt binder.

Technical Index	Unit	Test Results
Penetration (25 °C, 100 g, 5 s)	0.1 mm	25.6
Softening point, T_R&B_	°C	59.7
Ductility (15 °C, 5 cm/min)	cm	6.3
Ductility (10 °C, 5 cm/min)	cm	0
Kinematic viscosity @135 °C	Pa·s	0.913

Legend: T = temperature; R&B = ring and ball test.

**Table 3:** Technical properties of the soybean oil.

Technical Index	Unit	Test Results
Potential of hydrogen, pH	/	7 ± 0.5
Density	g/cm^3^	0.923
Dynamic viscosity @60 °C	Pa·s	0.16
Dielectric constant	F/m	2.8
Flash point	°C	234

**Table 4:** The elemental components of bio-oil, base asphalt binder, and BRAA.

Element Contents	C/%	O/%	H/%	N/%	S/%	H/C
Bio-oil	77.14	11.43	11.43	-	-	1.78
BRAA (4.0%)	74.86	10.50	12.95	0.48	0.20	2.0
A-70# base asphalt binder	81.6	0.9	10.8	0.77	0.68	1.51

Legend: C = carbon, H = hydrogen, N = nitrogen, O = oxygen, S = sulfur.

**Table 5:** Diffusion coefficients of the BRAA components.

Bio-Oil Content (%wt)	D_Asphaltenes_ (cm^2^/s)	D_Saturates_ (cm^2^/s)	D_Resins_ (cm^2^/s)	D_Aromatics_ (cm^2^/s)	D_Bio-oil_ (cm^2^/s)
1.0	7.67 × 10^−9^	4.17 × 10^−9^	1.48 × 10^−8^	7.00 × 10^−9^	6.03 × 10^−8^
2.0	7.00 × 10^−9^	7.50 × 10^−9^	1.63 × 10^−8^	8.50 × 10^−9^	2.07 × 10^−8^
3.0	6.00 × 10^−9^	7.83 × 10^−9^	1.38 × 10^−8^	8.67 × 10^−9^	4.70 × 10^−8^
4.0	1.52 × 10^−8^	1.33 × 10^−8^	3.47 × 10^−8^	4.82 × 10^−8^	3.92 × 10^−8^
5.0	1.67 × 10^−8^	1.39 × 10^−8^	3.51 × 10^−8^	4.99 × 10^−8^	4.41 × 10^−8^

**Table 6:** Diffusion coefficients at different temperatures.

4.0 wt. % Bio-Oil Content (%)	D_Asphaltenes_ (cm^2^/s)	D_Saturates_ (cm^2^/s)	D_Resins_ (cm^2^/s)	D_Aromatics_ (cm^2^/s)	D_Bio-oil_ (cm^2^/s)
D_273 K_	1.10 × 10^−8^	7.50 × 10^−9^	1.87 × 10^−8^	1.66 × 10^−8^	3.62 × 10^−8^
D_298 K_	1.52 × 10^−8^	1.33 × 10^−8^	3.47 × 10^−8^	4.82 × 10^−8^	3.92 × 10^−8^
D_318 K_	1.68 × 10^−8^	1.27 × 10^−8^	3.03 × 10^−8^	5.05 × 10^−8^	5.32 × 10^−8^
D_333 K_	7.71 × 10^−9^	6.67 × 10^−9^	7.67 × 10^−9^	7.50 × 10^−9^	8.33 × 10^−9^
D_353 K_	1.13 × 10^−8^	1.33 × 10^−8^	1.78 × 10^−8^	2.30 × 10^−8^	1.73 × 10^−8^

**Table 7:** Solubility parameter (*δ*) results.

Material Component	Solubility (*δ*)	|*A* − *B*|	ΔS*
Bio-oil (soybean)	16.582	Bio-oil–Asphaltenes	0.340
Asphaltenes	17.027	Bio-oil–Saturates	1.945
Saturates	14.742	Bio-oil–Resins	0.357
Aromatics	16.330	Bio-oil–Aromatics	0.316
Resins	16.371	Bio-oil–Asphalt binder	0.405
Asphalt binder	16.282	Bio-oil–Aged asphalt binder	0.550
Aged asphalt binder	17.237		

Legend: ΔS* represents the difference in quantity; Δ*S* is the absolute value of *A* minus *B*.

## Data Availability

The data presented in this study are available on request from the corresponding author.

## Sample Availability

Samples of the synthetic are available from the authors upon request.
